# Differential Expression and Bioinformatic Analysis of the circRNA Expression in Migraine Patients

**DOI:** 10.1155/2020/4710780

**Published:** 2020-10-07

**Authors:** Jinghan Lin, Shanshan Shi, Qihui Chen, Yonghui Pan

**Affiliations:** Department of Neurology, First Affiliated Hospital of Harbin Medical University, Postal street 23, Harbin, China

## Abstract

**Background:**

CircRNAs are noncoding RNA molecules that have recently been described and shown to regulate miRNA functionality. While recent studies have suggested such circRNAs to be associated with pain related diseases in humans, no comprehensive migraine-related circRNA profiles have been generated, and there is currently no clear understanding of whether they can serve as regulators of migraine pathology.

**Methods:**

We initially conducted a circRNA microarray analysis of the plasma of migraine patients and healthy controls. Based upon these data, we then selected 8 differentially expressed circRNAs and confirmed their expression in more migraine patient plasma samples via real-time PCR. We then performed functional and pathway enrichment analyses. Lastly, using a robust rank aggregation approach, we constructed a ceRNA network according to predicted circRNA–miRNA and miRNA–mRNA pairs in these migraine patient samples.

**Results:**

We were able to detect 2039 circRNAs in our patient samples, with 794 of 1245 these circRNAs being up- and downregulated in migraine patients relative to controls, respectively (fold change ≥ 1.5, *p* < 0.01). A qRT-PCR analysis confirmed that the expression of hsa_circRNA_100236, hsa_circRNA_102413, and hsa_circRNA_000367 was significantly enhanced in migraine patients, whereas the expression of hsa_circRNA_103809, hsa_circRNA_103670, and hsa_circRNA_101833 was significantly reduced in these individuals relative to healthy controls. We found these differentially regulated circRNAs to be associated with numerous predicted biological processes, with enrichment analyses suggesting that they may modulate the PI3K-Akt signaling so as to promote inflammation to drive migraine development. However, further research will be needed to formally test these mechanistic possibilities and to validate these circRNAs as potential biomarkers of migraine patients.

**Conclusions:**

Our results offer new potential insights into the mechanistic basis of this condition and suggest that hsa_circRNA_000367 and hsa_circRNA_102413 may offer value as regulators of migraine pathology.

## 1. Introduction

Migraines are a very common form of primary headache, typically presenting as unilateral headaches of moderate to severe intensity. Migraine patients frequently suffer from frequent and potentially debilitating attacks, which can also be accompanied by photophobia, nausea, and sensitivity to noise. Migraines are estimated to affect 10-20% of individuals, with incidence peaking in young adults and middle-aged individuals, in whom these headaches can markedly reduce productivity and quality of life [1]. The World Health Organization has classified migraine headaches as one of the most serious chronic functional disorders globally, highlighting the clear unmet medical need pertaining to its management and treatment [2]. As migraine pathogenesis is complex and not fully understood, at present, it can be very difficult to accurately diagnose, thus limiting the ability of clinicians to accurately treat patients or to make appropriate prognostic determinations regarding their condition. In an effort to identify more reliable biomarkers of migraine, recent studies have determined that migraine patients exhibit patterns of differential microRNA (miRNA) expression [3–6], with miRNAs also serving to modulate epigenetics in the context of chronic neuralgia [7, 8] and migraine [9, 10]. These miRNAs have also been found to provide functional insights into patient drug exposure [11], allowing for the identification of specific miRNA biomarkers of a given patient population or condition [12]. Recent studies have identified circular RNAs (circRNAs) as novel posttranscriptional regulators of the miRNA activity that can control diverse processes including transcription, mRNA splicing, translation, and RNA degradation. These circRNAs are generated through specific and selective splicing events, and they are expressed at high levels within eukaryotic cells. Although first detected within RNA viruses [13], RNA-sequencing studies have more recently highlighted the widespread expression of circRNAs associated with a wide range of human genes [14, 15]. Importantly, these circRNAs can serve as sponges for specific target miRNAs, binding, and sequestering them and thereby preventing them from executing their normal regulatory functions within cells. In addition, as they are closed loops of RNA, circRNAs are highly resistant to the RNA exonuclease activity. Given their stability, circRNAs are thus potentially ideal as novel diagnostic biomarkers of a range of different conditions. No studies to date, however, have conducted exhaustive circRNA profiling in order to understand whether the circRNA expression and/or activity are associated with migraine pathogenesis.

In the present study, we therefore utilized a high-throughput sequencing approach in an effort to identify differentially expressed circRNAs in migraine patients, and we then performed appropriate bioinformatic analyses on the identified circRNAs. Based on their differential expression profiles, we were able to identify specific migraine-associated circRNAs, with qRT-PCR being used to formally confirm their migraine-specific expression patterns in patient plasma samples. In addition, we conducted GO and KEGG enrichment analyses in order to understand the functionality of these circRNAs, and we constructed a ceRNA network in order to begin to elucidate the mechanisms whereby these circRNAs may influence the migraine onset. Together, the results of this study will serve as a foundation for future research regarding biomarkers and mechanisms of migraine pathology.

## 2. Materials and Methods

### 2.1. Sample Collection

We obtained peripheral blood samples for 4 migraine patients and 3 healthy control patients. All samples were collected from outpatients at the First Affiliated Hospital of Harbin Medical University (Harbin, China). This study was designed so as to conform with the 1964 revision of the Helsinki Declaration and was approved by the Ethics Committee of the First Affiliated Hospital of Harbin Medical University. All patients provided written informed consent. Patients were diagnosed with migraines based on the 3rd edition of the headache classification standard developed by IHS. Clinical data for these four patients are compiled in Supplementary Table 1. We took peripheral blood samples from patients who were between migraine attacks.

### 2.2. Differential circRNA Differential Expression Analysis

We have completed the Arraystar Human circRNA Array V2 analysis of the 7 samples. Total RNA from each sample was quantified using the NanoDrop ND-1000. The sample preparation and microarray hybridization were performed based on the Arraystar's standard protocols. Briefly, total RNAs were digested with Rnase R (Epicentre, Inc.) to remove linear RNAs and enrich circular RNAs. Then, the enriched circular RNAs were amplified and transcribed into fluorescent cRNA utilizing a random priming method (Arraystar Super RNA Labeling Kit; Arraystar). The labeled cRNAs were hybridized onto the Arraystar Human circRNA Array V2 (8x15K, Arraystar). After having washed the slides, the arrays were scanned by the Agilent Scanner G2505C. Agilent Feature Extraction software (version 11.0.1.1) was used to analyze acquired array images. Quantile normalization and subsequent data processing were performed using the R software limma package. Differentially expressed circRNAs with statistical significance between two groups were identified through Volcano Plot filtering. Differentially expressed circRNAs between two samples were identified through fold change filtering. Hierarchical clustering was performed to show the distinguishable circRNA expression pattern among samples.

### 2.3. qRT-PCR

After total RNA was isolated from individual samples, SuperScript III Reverse Transcriptase (Thermo Fisher Scientific) was used to prepare cDNA samples via reverse transcription. We then confirmed the differential expression of 7 different circRNAs in these donor samples using FastStart Universal SYBR Green Master (Rox) with the specific primers detailed in Table 1. Thermocycler settings were 95°C for 10 min, 40 cycles of 95°C for 10 s, and 60°C for 1 min. GAPDH served as a reference gene, and all samples were assayed in triplicate. The 2 − *ΔΔ*Ct method was used to assess the relative circRNA expression, with the circRNA expression in migraine patients being represented as fold change over control.

### 2.4. Pathway and Functional Enrichment Analyses

The Gene Ontology project provides a controlled vocabulary to describe gene and gene product attributes in any organism (http://www.geneontology.org). The ontology covers three domains: biological process, cellular component, and molecular function. Fisher's exact test in Bioconductor's top GO is used to find if there is more overlap between the DE list and the GO annotation list than would be expected by chance. The *p* value produced by top GO denotes the significance of GO terms enrichment in the DE genes. The lower the *p* value, the more significant the GO Term (*p* value≤0.05 is recommended). The pathway analysis is a functional analysis mapping gene to KEGG pathways. The *p* value (EASE score, Fisher *p* value, or Hypergeometric *p* value) denotes the significance of the pathway correlated to the conditions. The lower the *p* value, more significant is the pathway. (the recommend *p* value cut-off is 0.05).

### 2.5. CircRNA-Related ceRNA Regulatory Network Construction

CeRNA hypothesis RNA transcripts can crosstalk by competing for common microRNAs, with microRNA response elements (MREs) as the foundation of this interaction [16]. These RNA transcripts have been termed as competing endogenous RNAs–ceRNAs [17]. Any RNA transcripts with MREs might act as ceRNA, and ceRNAs include pseudogene transcripts, lncRNAs, circRNAs, and mRNAs; these transcripts can compete for the same microRNA response elements (MERs) to regulate mutually. To find the potential target of microRNAs, the target/microRNAs is predicted with home-made miRNA target prediction software based on TargetScan & miRanda [18–20].Through merging the common targeted miRNAs, we constructed the ceRNA network. There are three conditions that must exist for the ceRNA network to occur [16]. First, the relative concentration of the ceRNAs and their microRNAs is clearly important; second, the effectiveness of a ceRNA would depend on the number of microRNAs that it can “sponge”; third, not all of the MREs on ceRNAs are equal. So, we only accept these ceRNA-pair relations passing some measures filtering. Besides a measure with the number of common microRNAs, a hypergeometric test is executed for each ceRNA pair separately, which is defined by four parameters: (i) *N* is the total number of miRNAs used to predict targets; (ii) *K* is the number of miRNAs that interact with the chosen gene of interest; (iii) *n* is the number of miRNAs that interact with the candidate ceRNA of the chosen gene; and (iv) *i* is the common miRNA number between the two genes. The test calculates the *p* value by using the following formula:

(1) $$\begin{array}{c} P=\overset{\min\left(K,n\right)}{\underset{i=c}{\sum }}\frac{\left(\underset{i}{K}\right)\left(\underset{n-i}{N-K}\right)}{\underset{n}{N}} \end{array}$$

## 3. Results

### 3.1. Identification of circRNAs Differentially Expressed in Migraine Patients

**Figure 1:**
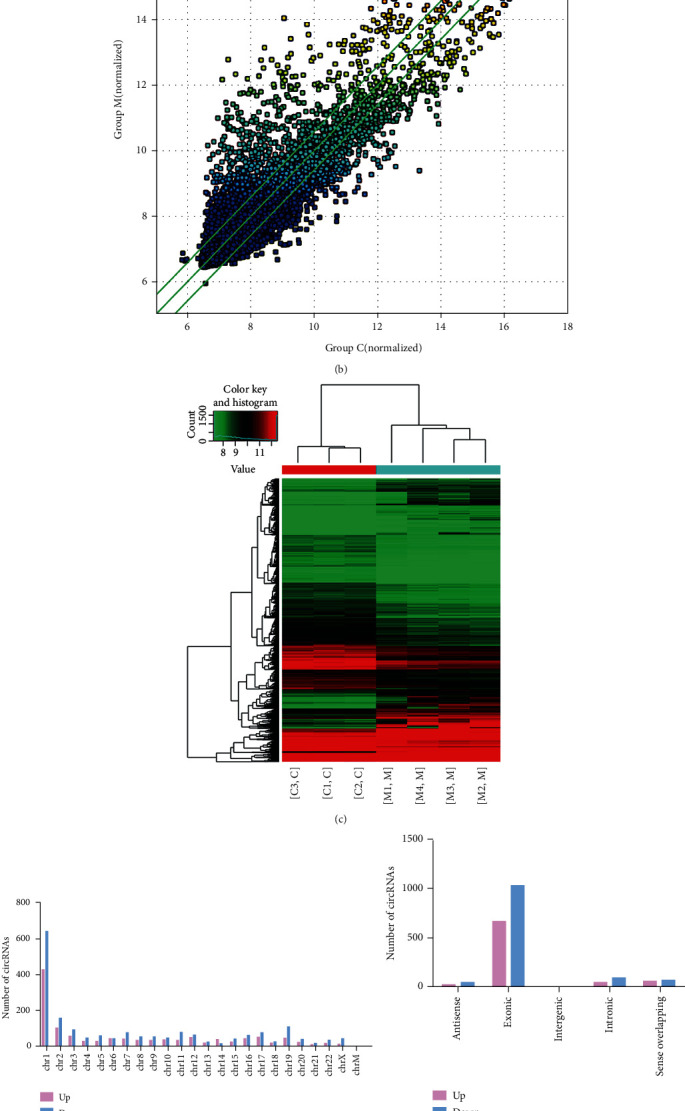
Identification of migraine-associated circRNA expression profiles and qRT-PCR confirmation of differential circRNA expression profiles. Differentially expressed circRNAs are shown in a volcano plot (a) and a scatter plot (b). Those circRNAs that were up- and downregulated by at least twofold in migraine patients relative to controls are shown in red, respectively (*p* < 0.05). Gray dots correspond to circRNAs for which no differential expression was observed. (c) Hierarchical clustering of these circRNAs. CircRNAs that were differentially expressed in the plasma of migraine patients relative to healthy controls are shown with corresponding chromosomal locations. While circRNAs were identified corresponding to all chromosomes, many were concentrated on chromosomes 1 and 2 (d). CircRNA types are shown, with may being derived from protein-coding exons, some being derived from introns, and a few from intergenic regions (e).The relative expression of the 8 indicated circRNAs in migraine and control patient samples, as assessed via microarray (f) and qRT-PCR (g). The expression was normalized to control samples. *n* = 3 samples per group. Data are means with SD. ^∗∗^*p* < 0.01, Student's *t*-test.

We first undertook a high-throughput circRNA microarray approach in order to determine which circRNAs were differentially expressed in migraine patients, analyzing blood samples from 4 migraine patients and 3 healthy donors. This analysis identified 13,209 total circRNAs in these samples distributed across all chromosomes, with the greatest proportion coming from chromosome 1 (Figure 1(d)). From a classification perspective, the majority of these circRNAs were derived from protein-coding exons, with others coming from introns, intergenic regions, and antisense regions (Figure 1(e)). An EdgeR analysis revealed that 2,039 circRNAs were differentially expressed in migraine patients (FC ≥ 1.5, *p* < 0.05), including 794 and 1245 that were up- and downregulated, respectively. Clear differences in the circRNA expression between control and migraine patients were evident in volcano plots (Figure 1(a)), scatter plots (Figure 1(b)), and hierarchical clustering analyses (Figure 1(c)), with the top 10 differentially regulated circRNAs shown in Table 1.

### 3.2. Confirmation of the Differential circRNA Expression

We next sought to validate the observed differential circRNA expression profiles in migraine patients by selecting 8 circRNAs for qRT-PCR analysis. Specifically, we selected 4 up- and 4 downregulated circRNAs, with results shown in Figures 1(f) and 1(g). This analysis confirmed the hsa_circRNA_103670 upregulation (3.56x), hsa_circRNA_103809 upregulation (2.03x), hsa_circRNA_101833 upregulation (1.34x), hsa_circRNA_000367 downregulation (0.47x), hsa_circRNA_100236 downregulation (0.73x), and hsa_circRNA_102413 downregulation (0.47x). These results thus confirmed that the results of our circRNA microarray analysis were accurate and highly reliable.

### 3.3. GO and KEGG Pathways Analysis of Differentially Expressed circRNAs

**Figure 2:**
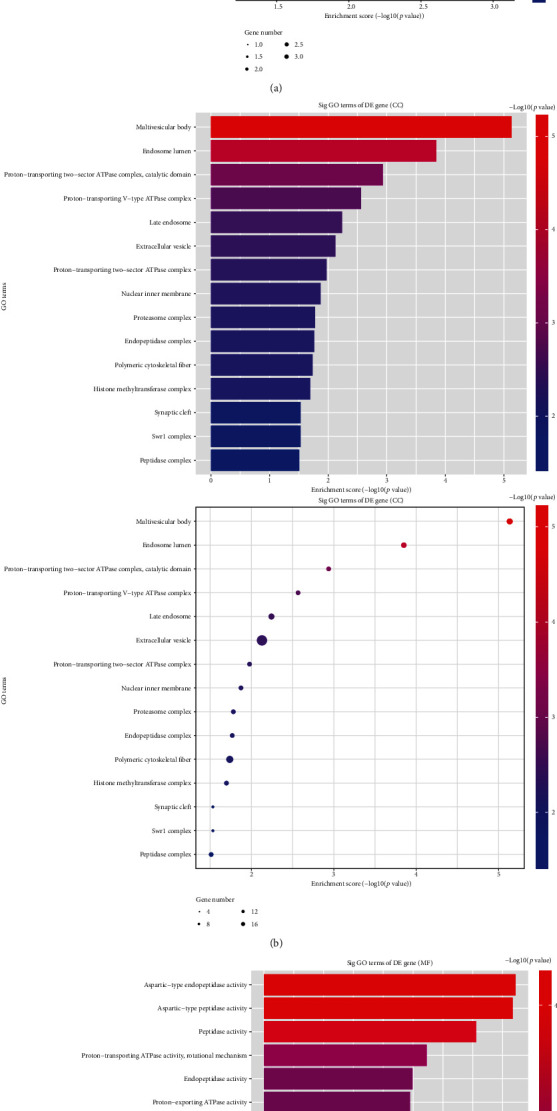
GO functional enrichment analysis. (a–c) GO functional enrichment analyses were performed on the genes corresponding to differentially expressed circRNA transcripts. GO terms are shown together with counts corresponding to the number of differentially expressed genes associated with that term. The black line corresponds to the −log10 value (*p* value). The enrichment factor corresponds to the ratio of the number of differentially enriched genes in a given pathway relative to the total number of genes in that pathway, with higher values corresponding to a higher degree of enrichment.

**Figure 3:**
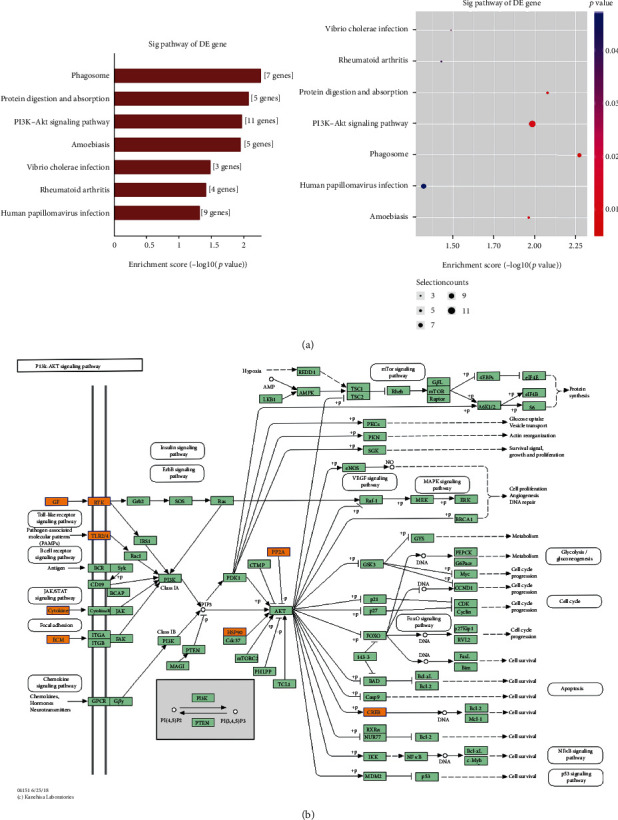
KEGG pathway enrichment analysis. (a) KEGG pathway enrichment analysis. A value of −log10 (*p* value) was used to gauge enrichment, with higher values corresponding to more significant enrichment. Gene numbers correspond to the number of genes enriched in a given pathway. The enrichment factor corresponds to the ratio of the number of differentially enriched genes in a given pathway relative to the total number of genes in that pathway, with higher values corresponding to a higher degree of enrichment. (b) The genes associated with migraine in the PI3K/Akt signaling pathway are shown.

We choose hsa_circRNA_103670, hsa_circRNA_103809, hsa_circRNA_000367, and hsa_circRNA_102413 for analysis. We conducted GO and KEGG analyses for the mRNA molecules corresponding to differentially expressed circRNAs in migraine patients. This analysis revealed these circRNAs to exhibit significant enrichment for 103 GO biological processes, 38 GO cellular components, 29 GO molecular functions, and 3 KEGG pathways. The most significantly enriched biological process- (BP-) related terms included “nerve growth factor signaling pathway”, “regulation of nonmotile cilium assembly”, and “pro-B cell differentiation” (Figure 2(a)). The most enriched cellular component- (CC-) related terms included “synaptic cleft”, “preribosome and small subunit precursor”, and “aminoacyl-tRNA synthetase multienzyme complex” (Figure 2(b)). The most enriched molecular function- (MF-) related terms included “oxidoreductase activity, acting on CH or CH2 groups”, “interleukin-1 receptor binding”, and “proton-exporting ATPase activity and phosphorylative mechanism” (Figure 2(c)). The KEGG pathway analyses revealed the PI3K-Akt signaling to be among those significantly migraine-related (Figure 3).

### 3.4. Construction of a ceRNA Network

**Figure 4:**
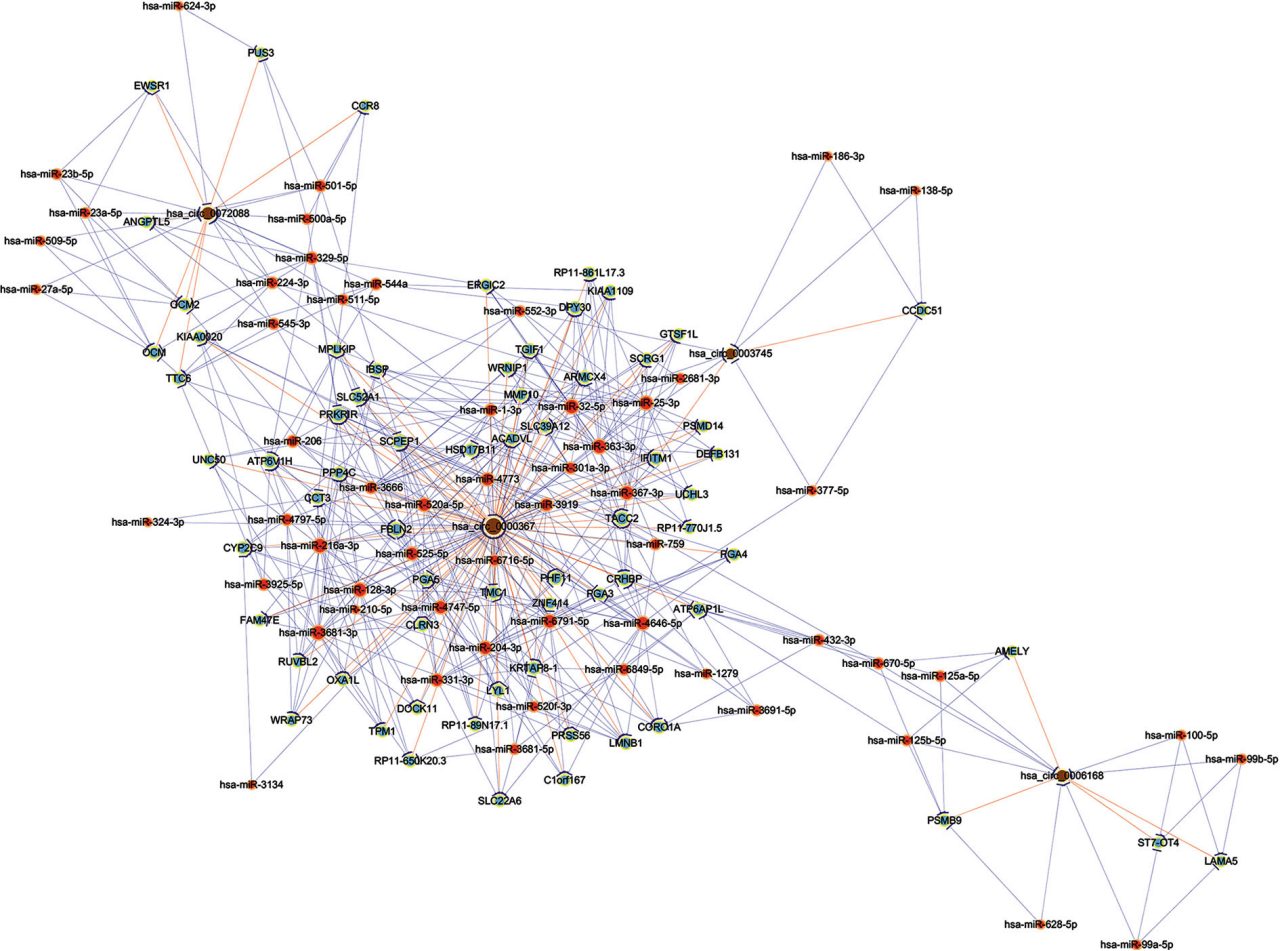
CircRNA and miRNA/gene network diagram. Shown is a circRNA–miRNA gene network diagram. Yellow, red, and blue nodes correspond to circRNAs, miRNAs, and genes, respectively, with lines indicating interactions between these entities.

We choose hsa_circRNA_103670, hsa_circRNA_103809, hsa_circRNA_000367, and hsa_circRNA_102413 for analysis. CircRNAs have been found to be capable of regulating the gene expression at least in part via functioning as sponges that sequester specific target miRNAs. In order to explore the potential regulatory role of the identified differentially expressed circRNAs in migraine patients, we therefore used the Targetscan and miRanda applications in order to predict the miRNA targets for these circRNAs as well as the mRNA targets downstream of these miRNAs. The final miRNA–gene relationship included 57 miRNAs and 66 genes. We then constructed a ceRNA network based upon this circRNA-miRNA-mRNA coregulatory model (Figure 4). The results of this network suggested that these circRNAs have many miRNA binding sites and thus have the potential to broadly regulate the gene expression. Additional data pertaining to this network is shown in Supplementary Table 2.

## 4. Discussion

There has been extensive research regarding circRNAs in recent years, with regulatory roles for these RNA molecules having been detected in the context of tumor formation [21], Parkinson's disease [22], Alzheimer's disease [23], and ischemic cerebrovascular disease [24]. No studies to date, however, have examined how circRNAs are associated with migraine pathogenesis. To that end, we conducted a microarray analysis to identify those circRNAs which were differentially abundant in circulation in migraine patients. In total, we detected 13,209 circRNAs in our patient samples, including 794 and 1245 that were up- and downregulated in migraine patients, respectively. We then further selected 8 differentially expressed circRNAs in order to validate their differential expression between migraine patients and healthy controls. This analysis confirmed that the expression patterns for hsa_circRNA_103670, hsa_circRNA_103809, hsa_circRNA_000367, and hsa_circRNA_102413 were consistent with our microarray findings.

We additionally conducted GO and KEGG analyses in order to understand the potential functional implications of these differentially expressed circRNAs. We found these circRNAs to be associated with GO terms including “pro-B cell differentiation”, “regulation of nonmotile cilium assembly”, “nerve growth factor signaling pathway”, “positive regulation of inositol phosphate biosynthetic process”, “synaptic cleft”, “aminoacyl-tRNA synthetase multienzyme complex”, “vacuolar proton-transporting V-type ATPase complex”, and “interleukin-1 receptor binding”. Furthermore, these circRNAs were significantly enriched for the PI3K/Akt signaling pathway.

At present, the mechanistic basis of migraine development is incompletely understood, with trigeminal nerve, vascular reflex, and cortical diffuse inhibition activities all being postulated to be involved in this process [25]. Neurogenic inflammation can occur when meningeal notional receptor peripheral terminals depolarize, leading to the trigeminal nerve and vascular system activation and the release of vasoactive peptides including calcitonin gene-related peptide (CGRP), which can cause dural vasodilation, increased intracranial blood flow, plasma protein extravasation, mast cell degranulation, and inflammation [26]. This, in turn, leads to increased primary neuron excitability in the trigeminal ganglion, as well as secondary and tertiary neuron sensitization, resulting in pain. Both inflammatory factors such as IL-1, IL-6, TNF alpha, and TGF beta, as well as anti-inflammatory factors including IL-4, IL-10, and IL-13 are related to this process [27]. Deng et al. previously found CGRP to induce the upregulation of Ithe L-6 mRNA expression via mmu_circRNA_007893, which served as a sponge for the miRNA mmu-mir-485-5p [28]. The IL-1 cytokine family is composed of 11 proteins, among which IL-1 beta has a strong proinflammatory effect and is an important mediator mediating the immune response[29]. IL-1 beta levels have been shown to be increased in the circulation of migraine patients and in the dural membrane of migraine model rats [30, 31]. Han et al. also observed significant increases in circulating IL-6, IL-1 beta, and TNF levels in migraine patients relative to healthy controls [32]. This raises the possibility that circRNAs may regulate IL-6, IL-1 beta, and other inflammatory factors in order to influence migraine development. The PI3K/Akt signaling has also been shown to be active in the brain tissue of migraine model rats. When tensin homolog proteins upstream of the PI3K/Akt signaling pathway, this can lead to enhanced PIP 3, 4, and 5 activation, leading to glycogen synthase kinase-3 beta inhibition [33]. We hypothesize that certain differentially expressed circRNAs identified in our analysis (hsa_circRNA_103670, hsa_circRNA_103809, hsa_circRNA_000367, hsa_circRNA_ 102413) may modulate the PI3K/Akt signaling and consequent production of IL-1 beta, CGRP, or other factors leading to migraine development.

Many recent studies have explored the functionality of ceRNAs, revealing them to be capable of functioning by binding to MREs, thereby releasing miRNAs from the RISC complex and preventing the degradation of their target mRNAs, leading to the corresponding gene upregulation [34]. There are multiple classes of ceRNAs, including lncRNAs, pseudogenes, and circRNAs. CircRNAs contain miRNA binding sites, allowing these circRNAs to serve as molecular sponges for specific miRNAs, thereby influencing the expression of their downstream target mRNA molecules, indirectly influencing cellular biology [14, 35]. Such circRNA regulatory functions have been shown to influence the incidence of Alzheimer's disease and ischemic cerebrovascular disease [32, 36]. Their role in the context of migraine development, however, has not been previously explored. The hsa_circRNA_000367 circRNA has been found to regulate the TGIF1 (inhibiting transformed growth factor-beta signal), IFITM1 (interferon-induced transmembrane protein 1), and PRKRIR (PRKRIR protein kinase, interferon-induced double-stranded RNA-dependent inhibitor) genes via a ceRNA mechanism, with hsa_circRNA_102413 also regulating IFITM1. The specific expression patterns of MCP-1, IL-10, TGF-beta, and other cytokines have also been linked with migraine disease development [37, 38]. Furthermore, Saygi et al. suggested that TGF-beta 1 genotypes are associated with pediatric migraine development [39]. This raises the possibility that hsa_circRNA_000367 and hsa_circRNA_102413 may modulate migraine pathogenesis via regulating the expression of these and other inflammatory factors, although further experiments will be needed to confirm this possibility.

## 5. Conclusions

In conclusion, our study identified a number of circRNAs that were differentially expressed in migraine patients. We further confirmed the differential expression of specific circRNAs (hsa_circRNA_103670, hsa_circRNA_103809, hsa_circRNA_ 000367, hsa_circRNA_102413), and further bioinformatics analyses revealed these circRNAs to be associated with migraine development. However, further experiments will be necessary in order to confirm the relationship between these circRNAs and migraine development. Even so, our findings can serve as a foundation for future studies of migraine biomarkers and functional regulators.

## Figures and Tables

**Table 1:** The top 10 up- and downregulated differentially expressed circRNAs in migraine patients. FC: fold change relative to control; MRE: miRNA response element.

circRNA	Alias	Chrom	*p* value	FC (abs)	Regulation	circRNA_type	Gene symbol	MRE1	MRE2	MRE3	MRE4	MRE5
hsa_circRNA_103670	hsa_circ_0006168	chr4	0.000005335	66.9668	Up	Exonic	CNOT6L	Hsa-miR-570-5p	Hsa-miR-592	Hsa-miR-542-3p	Hsa-miR-628-5p	Hsa-miR-148b-5p
hsa_circRNA_101833	hsa_circ_0039908	chr16	0.000193084	14.1092	Up	Exonic	DUS2	Hsa-miR-590-5p	Hsa-miR-21-5p	Hsa-miR-654-3p	Hsa-miR-640	Hsa-miR-572
hsa_circRNA_103809	hsa_circ_0072088	chr5	0.000178409	13.6281	Up	Exonic	ZFR	Hsa-miR-511-5p	Hsa-miR-130b-5p	Hsa-miR-642a-5p	Hsa-miR-532-3p	Hsa-miR-329-5p
hsa_circRNA_104855	hsa_circ_0087888	chr9	0.013291027	12.7997	Up	Exonic	TMEM245	Hsa-miR-567	Hsa-miR-183-5p	Hsa-miR-652-3p	Hsa-let-7f-1-3p	Hsa-miR-338-5p
hsa_circRNA_104761	hsa_circ_0001847	chr9	0.001438757	11.1789	Up	Exonic	UBAP2	Hsa-miR-34c-5p	Hsa-miR-449a	Hsa-miR-449b-5p	Hsa-miR-449c-5p	Hsa-miR-370-3p
hsa_circRNA_102610	hsa_circ_0000972	chr2	0.006983514	9.7333	Up	Exonic	MBOAT2	Hsa-miR-330-3p	Hsa-miR-130b-3p	Hsa-miR-130a-3p	Hsa-miR-513a-3p	Hsa-miR-136-5p
hsa_circRNA_103444	hsa_circ_0008797	chr3	0.048378398	8.4084	Up	Exonic	GSK3B	Hsa-miR-508-5p	Hsa-miR-500a-5p	Hsa-miR-7-5p	Hsa-miR-29a-5p	Hsa-miR-623
hsa_circRNA_100257	hsa_circ_0002454	chr1	0.008199393	8.4059	Up	Exonic	DNAJC6	Hsa-miR-508-3p	Hsa-miR-542-3p	Hsa-miR-516b-5p	Hsa-miR-518e-5p	Hsa-miR-519b-5p
hsa_circRNA_103149	hsa_circ_0002903	chr21	0.004270572	7.7418	Up	Exonic	PCNT	Hsa-miR-29b-1-5p	Hsa-miR-329-5p	Hsa-miR-136-5p	Hsa-miR-1264	Hsa-miR-26b-3p
hsa_circRNA_100983	hsa_circ_0024766	chr11	0.026330597	7.2345	Up	Exonic	STT3A	Hsa-miR-376a-2-5p	Hsa-miR-873-5p	Hsa-miR-765	Hsa-miR-576-3p	Hsa-miR-423-3p
hsa_circRNA_000367	hsa_circ_0000367	chr11	0.000764274	15.1939	Down	Exonic	SIAE	Hsa-miR-331-3p	Hsa-miR-4646-5p	Hsa-miR-4797-5p	Hsa-miR-3919	Hsa-miR-3190-3p
hsa_circRNA_100236	hsa_circ_0012634	chr1	0.000005729	6.6553	Down	Exonic	TMEM59	Hsa-miR-508-5p	Hsa-miR-372-5p	Hsa-miR-655-5p	Hsa-miR-519d-5p	Hsa-miR-668-3p
hsa_circRNA_100790	hsa_circ_0000288	chr11	0.005757781	5.7756	Down	Exonic	CAPRIN1	Hsa-miR-20b-3p	Hsa-miR-150-3p	Hsa-miR-133a-5p	Hsa-miR-509-3p	Hsa-miR-485-5p
hsa_circRNA_100789	hsa_circ_0021652	chr11	0.004277333	5.6822	Down	Exonic	CAPRIN1	Hsa-miR-873-5p	Hsa-miR-20b-3p	Hsa-miR-649	Hsa-miR-150-3p	Hsa-miR-133a-5p
hsa_circRNA_102413	hsa_circ_0003745	chr19	0.000034324	5.4179	Down	Exonic	PIP5K1C	Hsa-miR-432-5p	Hsa-miR-138-5p	Hsa-miR-1264	Hsa-miR-658	Hsa-miR-873-5p
hsa_circRNA_103689	hsa_circ_0007324	chr4	0.000103109	4.3421	Down	Exonic	PTPN13	Hsa-miR-548d-3p	Hsa-miR-489-3p	Hsa-miR-145-5p	Hsa-miR-29a-5p	Hsa-miR-520 g-5p
hsa_circRNA_101784	hsa_circ_0008223	chr16	0.000159064	4.1580	Down	Exonic	XPO6	Hsa-miR-106b-3p	Hsa-miR-500a-3p	Hsa-miR-412-3p	Hsa-miR-222-5p	Hsa-miR-638
hsa_circRNA_104950	hsa_circ_0089252	chr9	0.000552165	3.7261	Down	Exonic	RAPGEF1	Hsa-miR-766-3p	Hsa-miR-367-5p	Hsa-miR-9-5p	Hsa-miR-770-5p	Hsa-miR-504-5p
hsa_circRNA_103846	hsa_circ_0002512	chr5	0.000293167	3.6087	Down	Exonic	DEPDC1B	Hsa-miR-607	Hsa-miR-589-3p	Hsa-miR-605-5p	Hsa-miR-550a-5p	Hsa-miR-550a-3-5p
hsa_circRNA_101698	hsa_circ_0000669	chr16	0.000556995	3.3769	Down	Exonic	CARHSP1	Hsa-miR-185-3p	Hsa-miR-146b-3p	Hsa-miR-298	Hsa-miR-128-2-5p	Hsa-miR-92a-2-5p

## Data Availability

All data generated or analyzed during this study are included in this article.

## Supplementary Materials

Supplementary 1 Supplementary Table 1. Selected migraine patient clinical data.

Supplementary 2 Supplementary Table 2. The differentially expressed circRNA target microRNA and ceRNA network data.
