# Supplementary material for: Differential Expression and Bioinformatic Analysis of the circRNA Expression in Migraine Patients
Source: Biomed Res Int. 2020 Oct 7;2020:4710780. doi: 10.1155/2020/4710780 (PMC7607275; doi:10.1155/2020/4710780)
Supplement: Supplementary 1 — Supplementary Table 1. Selected migraine patient clinical data. [file 4710780.f1.doc]

**Supplementary Table 1**

Selected migraine patient clinical data.

| **name** | **Symptom** | **Time** | **Aura** | **Inducement** | **Frequency and duration** |
| --- | --- | --- | --- | --- | --- |
| **patient1** | Unilateral temporal head throbbing pain, occasionally accompanied by nausea and vomiting | 9 year | None | Poor sleep | Average 1 time/half a year  The duration of the attack varies from several hours to 24 hours |
| **patient2** | Throbbing pain in one side of the head | 5 year | None | None | Average 1-2 episodes per year, with episodes lasting from a few hours to 24 hours |
| **patient3** | Bilaterally or throughout the brain, swelling or throbbing pain | 5 year | None | Poor sleep，daytime fatigue | Average 1 time/half a year  The attack lasts from a few hours to 24 hours |
| **patient4** | Throbbing pain in one side of the head, accompanied by nausea, photophobia, and occasionally panic attacks | 10 year | None | Fatigue，depression | Average 1 time/half a year  The attack lasted several hours |
