# Supplementary material for: Differential Expression and Bioinformatic Analysis of the circRNA Expression in Migraine Patients
Source: Biomed Res Int. 2020 Oct 7;2020:4710780. doi: 10.1155/2020/4710780 (PMC7607275; doi:10.1155/2020/4710780)
Supplement: Supplementary 2 — Supplementary Table 2. The differentially expressed circRNA target microRNA and ceRNA network data. [file 4710780.f2.doc]

**Supplementary Table 2**

The differentially expressed circRNA target microRNA and ceRNA network data.

| **SeqName** | **GeneSymbol** | **GeneType** | **CeNames** | **CeSymbols** | **CeTypes** | **CommonMirnas** |
| --- | --- | --- | --- | --- | --- | --- |
| hsa_circRNA_103809 | hsa_circ_0072088 | circRNA | NM_001097622 | OCM | protein_coding | hsa-miR-329-5p,hsa-miR-509-5p,hsa-miR-23b-5p,hsa-miR-23a-5p,hsa-miR-27a-5p |
| hsa_circRNA_103809 | hsa_circ_0072088 | circRNA | NM_005243 | EWSR1 | protein_coding | hsa-miR-23b-5p,hsa-miR-224-3p,hsa-miR-23a-5p |
| hsa_circRNA_103809 | hsa_circ_0072088 | circRNA | NM_178127 | ANGPTL5 | protein_coding | hsa-miR-329-5p,hsa-miR-224-3p,hsa-miR-501-5p,hsa-miR-545-3p |
| hsa_circRNA_103809 | hsa_circ_0072088 | circRNA | NM_006188 | OCM2 | protein_coding | hsa-miR-329-5p,hsa-miR-509-5p,hsa-miR-23b-5p,hsa-miR-23a-5p,hsa-miR-27a-5p |
| hsa_circRNA_103809 | hsa_circ_0072088 | circRNA | NM_001310135 | TTC6 | protein_coding | hsa-miR-224-3p,hsa-miR-544a,hsa-miR-545-3p |
| hsa_circRNA_103809 | hsa_circ_0072088 | circRNA | NM_005201 | CCR8 | protein_coding | hsa-miR-511-5p,hsa-miR-501-5p,hsa-miR-500a-5p |
| hsa_circRNA_103809 | hsa_circ_0072088 | circRNA | NM_031307 | PUS3 | protein_coding | hsa-miR-624-3p,hsa-miR-501-5p,hsa-miR-500a-5p |
| hsa_circRNA_103670 | hsa_circ_0006168 | circRNA | UC021WFW.1 | LAMA5 | protein_coding | hsa-miR-99b-5p,hsa-miR-99a-5p,hsa-miR-100-5p |
| hsa_circRNA_103670 | hsa_circ_0006168 | circRNA | ENST00000397751 | ST7-OT4 | protein_coding | hsa-miR-99b-5p,hsa-miR-99a-5p,hsa-miR-100-5p |
| hsa_circRNA_103670 | hsa_circ_0006168 | circRNA | NM_002800 | PSMB9 | protein_coding | hsa-miR-628-5p,hsa-miR-125b-5p,hsa-miR-125a-5p,hsa-miR-670-5p |
| hsa_circRNA_103670 | hsa_circ_0006168 | circRNA | NM_001143 | AMELY | protein_coding | hsa-miR-125b-5p,hsa-miR-125a-5p,hsa-miR-670-5p |
| hsa_circRNA_102413 | hsa_circ_0003745 | circRNA | NM_003641 | IFITM1 | protein_coding | hsa-miR-552-3p,hsa-miR-32-5p,hsa-miR-363-3p |
| hsa_circRNA_102413 | hsa_circ_0003745 | circRNA | NM_024661 | CCDC51 | protein_coding | hsa-miR-138-5p,hsa-miR-377-5p,hsa-miR-186-3p |
| hsa_circRNA_000367 | hsa_circ_0000367 | circRNA | NM_000771 | CYP2C9 | protein_coding | hsa-miR-4797-5p,hsa-miR-4747-5p,hsa-miR-216a-3p,hsa-miR-128-3p,hsa-miR-3134,hsa-miR-3681-3p |
| hsa_circRNA_000367 | hsa_circ_0000367 | circRNA | NM_006997 | TACC2 | protein_coding | hsa-miR-4646-5p,hsa-miR-363-3p,hsa-miR-25-3p,hsa-miR-367-3p,hsa-miR-32-5p,hsa-miR-6791-5p,hsa-miR-1279,hsa-miR-204-3p |
| hsa_circRNA_000367 | hsa_circ_0000367 | circRNA | NM_007281 | SCRG1 | protein_coding | hsa-miR-363-3p,hsa-miR-25-3p,hsa-miR-367-3p,hsa-miR-32-5p |
| hsa_circRNA_000367 | hsa_circ_0000367 | circRNA | NM_001136570 | FAM47E | protein_coding | hsa-miR-3919,hsa-miR-216a-3p,hsa-miR-128-3p,hsa-miR-3681-3p |
| hsa_circRNA_000367 | hsa_circ_0000367 | circRNA | NM_001040443 | PHF11 | protein_coding | hsa-miR-4646-5p,hsa-miR-206,hsa-miR-1-3p,hsa-miR-4773,hsa-miR-2681-3p,hsa-miR-204-3p,hsa-miR-6716-5p |
| hsa_circRNA_000367 | hsa_circ_0000367 | circRNA | NM_004705 | PRKRIR | protein_coding | hsa-miR-3919,hsa-miR-520a-5p,hsa-miR-216a-3p,hsa-miR-206,hsa-miR-128-3p,hsa-miR-1-3p,hsa-miR-4773,hsa-miR-759,hsa-miR-525-5p,hsa-miR-3681-3p |
| hsa_circRNA_000367 | hsa_circ_0000367 | circRNA | NM_014878 | KIAA0020 | protein_coding | hsa-miR-4797-5p,hsa-miR-3919,hsa-miR-4773,hsa-miR-3925-5p |
| hsa_circRNA_000367 | hsa_circ_0000367 | circRNA | NM_170695 | TGIF1 | protein_coding | hsa-miR-363-3p,hsa-miR-4747-5p,hsa-miR-25-3p,hsa-miR-32-5p,hsa-miR-6716-5p,hsa-miR-3666 |
| hsa_circRNA_000367 | hsa_circ_0000367 | circRNA | NM_014224 | PGA5 | protein_coding | hsa-miR-331-3p,hsa-miR-4646-5p,hsa-miR-4773,hsa-miR-6791-5p,hsa-miR-204-3p |
| hsa_circRNA_000367 | hsa_circ_0000367 | circRNA | NM_021626 | SCPEP1 | protein_coding | hsa-miR-331-3p,hsa-miR-4646-5p,hsa-miR-520a-5p,hsa-miR-4747-5p,hsa-miR-520f-3p,hsa-miR-6791-5p,hsa-miR-204-3p,hsa-miR-525-5p |
| hsa_circRNA_000367 | hsa_circ_0000367 | circRNA | NM_014044 | UNC50 | protein_coding | hsa-miR-216a-3p,hsa-miR-206,hsa-miR-1-3p,hsa-miR-3681-3p |
| hsa_circRNA_000367 | hsa_circ_0000367 | circRNA | ENST00000534438 | RP11-770J1.5 | protein_coding | hsa-miR-363-3p,hsa-miR-25-3p,hsa-miR-367-3p,hsa-miR-32-5p |
| hsa_circRNA_000367 | hsa_circ_0000367 | circRNA | NM_144658 | DOCK11 | protein_coding | hsa-miR-216a-3p,hsa-miR-128-3p,hsa-miR-32-5p,hsa-miR-3681-3p |
| hsa_circRNA_000367 | hsa_circ_0000367 | circRNA | NM_017818 | WRAP73 | protein_coding | hsa-miR-4797-5p,hsa-miR-216a-3p,hsa-miR-128-3p,hsa-miR-3681-3p |
| hsa_circRNA_000367 | hsa_circ_0000367 | circRNA | NM_000018 | ACADVL | protein_coding | hsa-miR-4646-5p,hsa-miR-4747-5p,hsa-miR-216a-3p,hsa-miR-128-3p,hsa-miR-204-3p,hsa-miR-3681-3p |
| hsa_circRNA_000367 | hsa_circ_0000367 | circRNA | NM_005805 | PSMD14 | protein_coding | hsa-miR-363-3p,hsa-miR-25-3p,hsa-miR-367-3p,hsa-miR-32-5p |
| hsa_circRNA_000367 | hsa_circ_0000367 | circRNA | NM_015941 | ATP6V1H | protein_coding | hsa-miR-3919,hsa-miR-520a-5p,hsa-miR-216a-3p,hsa-miR-128-3p,hsa-miR-3925-5p,hsa-miR-525-5p,hsa-miR-3681-3p |
| hsa_circRNA_000367 | hsa_circ_0000367 | circRNA | NM_005998 | CCT3 | protein_coding | hsa-miR-216a-3p,hsa-miR-128-3p,hsa-miR-324-3p,hsa-miR-3681-3p |
| hsa_circRNA_000367 | hsa_circ_0000367 | circRNA | NM_001017971 | ATP6AP1L | protein_coding | hsa-miR-432-3p,hsa-miR-4773,hsa-miR-3681-5p,hsa-miR-6849-5p,hsa-miR-3691-5p |
| hsa_circRNA_000367 | hsa_circ_0000367 | circRNA | NM_002425 | MMP10 | protein_coding | hsa-miR-363-3p,hsa-miR-25-3p,hsa-miR-32-5p,hsa-miR-3666,hsa-miR-301a-3p |
| hsa_circRNA_000367 | hsa_circ_0000367 | circRNA | NM_006666 | RUVBL2 | protein_coding | hsa-miR-4797-5p,hsa-miR-216a-3p,hsa-miR-128-3p,hsa-miR-3681-3p |
| hsa_circRNA_000367 | hsa_circ_0000367 | circRNA | NM_020135 | WRNIP1 | protein_coding | hsa-miR-363-3p,hsa-miR-25-3p,hsa-miR-32-5p,hsa-miR-3666,hsa-miR-301a-3p |
| hsa_circRNA_000367 | hsa_circ_0000367 | circRNA | NM_015312 | KIAA1109 | protein_coding | hsa-miR-363-3p,hsa-miR-4773,hsa-miR-367-3p,hsa-miR-32-5p |
| hsa_circRNA_000367 | hsa_circ_0000367 | circRNA | NM_001145195 | SLC39A12 | protein_coding | hsa-miR-363-3p,hsa-miR-2681-3p,hsa-miR-32-5p,hsa-miR-3666,hsa-miR-301a-3p |
| hsa_circRNA_000367 | hsa_circ_0000367 | circRNA | NM_176791 | GTSF1L | protein_coding | hsa-miR-363-3p,hsa-miR-25-3p,hsa-miR-367-3p,hsa-miR-32-5p |
| hsa_circRNA_000367 | hsa_circ_0000367 | circRNA | NM_001040448 | DEFB131 | protein_coding | hsa-miR-363-3p,hsa-miR-25-3p,hsa-miR-367-3p,hsa-miR-32-5p |
| hsa_circRNA_000367 | hsa_circ_0000367 | circRNA | NM_002720 | PPP4C | protein_coding | hsa-miR-520a-5p,hsa-miR-216a-3p,hsa-miR-128-3p,hsa-miR-525-5p,hsa-miR-3681-3p |
| hsa_circRNA_000367 | hsa_circ_0000367 | circRNA | NM_138691 | TMC1 | protein_coding | hsa-miR-216a-3p,hsa-miR-128-3p,hsa-miR-4773,hsa-miR-520f-3p,hsa-miR-1279,hsa-miR-3681-3p |
| hsa_circRNA_000367 | hsa_circ_0000367 | circRNA | NM_032574 | DPY30 | protein_coding | hsa-miR-363-3p,hsa-miR-520a-5p,hsa-miR-2681-3p,hsa-miR-367-3p,hsa-miR-32-5p,hsa-miR-6716-5p |
| hsa_circRNA_000367 | hsa_circ_0000367 | circRNA | NM_001195129 | PRSS56 | protein_coding | hsa-miR-331-3p,hsa-miR-4646-5p,hsa-miR-6791-5p,hsa-miR-204-3p |
| hsa_circRNA_000367 | hsa_circ_0000367 | circRNA | NM_004790 | SLC22A6 | protein_coding | hsa-miR-331-3p,hsa-miR-4646-5p,hsa-miR-6791-5p,hsa-miR-204-3p |
| hsa_circRNA_000367 | hsa_circ_0000367 | circRNA | NM_032370 | ZNF414 | protein_coding | hsa-miR-331-3p,hsa-miR-4646-5p,hsa-miR-4747-5p,hsa-miR-6791-5p |
| hsa_circRNA_000367 | hsa_circ_0000367 | circRNA | NM_001010881 | C1orf167 | protein_coding | hsa-miR-4646-5p,hsa-miR-4747-5p,hsa-miR-6791-5p,hsa-miR-204-3p |
| hsa_circRNA_000367 | hsa_circ_0000367 | circRNA | NM_005015 | OXA1L | protein_coding | hsa-miR-4747-5p,hsa-miR-216a-3p,hsa-miR-128-3p,hsa-miR-3681-3p |
| hsa_circRNA_000367 | hsa_circ_0000367 | circRNA | NM_152311 | CLRN3 | protein_coding | hsa-miR-3919,hsa-miR-520a-5p,hsa-miR-216a-3p,hsa-miR-525-5p,hsa-miR-3681-3p |
| hsa_circRNA_000367 | hsa_circ_0000367 | circRNA | NM_001079808 | PGA4 | protein_coding | hsa-miR-331-3p,hsa-miR-4646-5p,hsa-miR-4773,hsa-miR-6791-5p,hsa-miR-204-3p |
| hsa_circRNA_000367 | hsa_circ_0000367 | circRNA | NM_007074 | CORO1A | protein_coding | hsa-miR-4646-5p,hsa-miR-432-3p,hsa-miR-6849-5p,hsa-miR-3691-5p,hsa-miR-204-3p,hsa-miR-6716-5p |
| hsa_circRNA_000367 | hsa_circ_0000367 | circRNA | NM_001998 | FBLN2 | protein_coding | hsa-miR-520a-5p,hsa-miR-216a-3p,hsa-miR-206,hsa-miR-128-3p,hsa-miR-1-3p,hsa-miR-6791-5p,hsa-miR-525-5p,hsa-miR-3681-3p |
| hsa_circRNA_000367 | hsa_circ_0000367 | circRNA | NM_016570 | ERGIC2 | protein_coding | hsa-miR-363-3p,hsa-miR-25-3p,hsa-miR-367-3p,hsa-miR-32-5p |
| hsa_circRNA_000367 | hsa_circ_0000367 | circRNA | NM_006002 | UCHL3 | protein_coding | hsa-miR-3919,hsa-miR-363-3p,hsa-miR-25-3p,hsa-miR-367-3p,hsa-miR-32-5p |
| hsa_circRNA_000367 | hsa_circ_0000367 | circRNA | NM_138701 | MPLKIP | protein_coding | hsa-miR-520a-5p,hsa-miR-206,hsa-miR-1-3p,hsa-miR-525-5p |
| hsa_circRNA_000367 | hsa_circ_0000367 | circRNA | NM_005573 | LMNB1 | protein_coding | hsa-miR-3919,hsa-miR-520a-5p,hsa-miR-216a-3p,hsa-miR-759,hsa-miR-520f-3p,hsa-miR-525-5p,hsa-miR-3681-3p |
| hsa_circRNA_000367 | hsa_circ_0000367 | circRNA | NM_001079807 | PGA3 | protein_coding | hsa-miR-331-3p,hsa-miR-4646-5p,hsa-miR-4773,hsa-miR-6791-5p,hsa-miR-204-3p |
| hsa_circRNA_000367 | hsa_circ_0000367 | circRNA | NM_001882 | CRHBP | protein_coding | hsa-miR-4646-5p,hsa-miR-363-3p,hsa-miR-432-3p,hsa-miR-25-3p,hsa-miR-367-3p,hsa-miR-32-5p,hsa-miR-3691-5p,hsa-miR-204-3p |
| hsa_circRNA_000367 | hsa_circ_0000367 | circRNA | ENST00000358290 | HSD17B11 | protein_coding | hsa-miR-4797-5p,hsa-miR-206,hsa-miR-1-3p,hsa-miR-4773,hsa-miR-759,hsa-miR-3666,hsa-miR-301a-3p |
| hsa_circRNA_000367 | hsa_circ_0000367 | circRNA | NM_017986 | SLC52A1 | protein_coding | hsa-miR-331-3p,hsa-miR-363-3p,hsa-miR-25-3p,hsa-miR-367-3p,hsa-miR-32-5p,hsa-miR-6791-5p |
| hsa_circRNA_000367 | hsa_circ_0000367 | circRNA | NM_175857 | KRTAP8-1 | protein_coding | hsa-miR-331-3p,hsa-miR-3919,hsa-miR-3681-5p,hsa-miR-3925-5p,hsa-miR-6849-5p,hsa-miR-6791-5p |
| hsa_circRNA_000367 | hsa_circ_0000367 | circRNA | ENST00000551650 | RP11-650K20.3 | protein_coding | hsa-miR-216a-3p,hsa-miR-128-3p,hsa-miR-3681-5p,hsa-miR-6849-5p,hsa-miR-3681-3p |
| hsa_circRNA_000367 | hsa_circ_0000367 | circRNA | NM_004967 | IBSP | protein_coding | hsa-miR-216a-3p,hsa-miR-25-3p,hsa-miR-367-3p,hsa-miR-32-5p,hsa-miR-3681-3p |
| hsa_circRNA_000367 | hsa_circ_0000367 | circRNA | NM_003641 | IFITM1 | protein_coding | hsa-miR-363-3p,hsa-miR-216a-3p,hsa-miR-128-3p,hsa-miR-367-3p,hsa-miR-32-5p |
| hsa_circRNA_000367 | hsa_circ_0000367 | circRNA | ENST00000579678 | RP11-861L17.3 | protein_coding | hsa-miR-363-3p,hsa-miR-25-3p,hsa-miR-367-3p,hsa-miR-32-5p |
| hsa_circRNA_000367 | hsa_circ_0000367 | circRNA | NM_005583 | LYL1 | protein_coding | hsa-miR-520a-5p,hsa-miR-4747-5p,hsa-miR-210-5p,hsa-miR-6791-5p,hsa-miR-525-5p |
| hsa_circRNA_000367 | hsa_circ_0000367 | circRNA | ENST00000311067 | RP11-89N17.1 | protein_coding | hsa-miR-3919,hsa-miR-520a-5p,hsa-miR-4747-5p,hsa-miR-525-5p |
| hsa_circRNA_000367 | hsa_circ_0000367 | circRNA | NM_001256155 | ARMCX4 | protein_coding | hsa-miR-520a-5p,hsa-miR-759,hsa-miR-525-5p,hsa-miR-3666,hsa-miR-301a-3p |
| hsa_circRNA_000367 | hsa_circ_0000367 | circRNA | NM_001018005 | TPM1 | protein_coding | hsa-miR-216a-3p,hsa-miR-128-3p,hsa-miR-3925-5p,hsa-miR-3681-3p |
